# Downregulated Dual-Specificity Protein Phosphatase 1 in Ovarian Carcinoma: A Comprehensive Study With Multiple Methods

**DOI:** 10.3389/pore.2022.1610404

**Published:** 2022-07-15

**Authors:** Zi-Qian Liang, Rong-Quan He, Jia-Yuan Luo, Zhi-Guang Huang, Jie Li, Lu-Yang Zhong, Jun-Hong Chen, Su-Ning Huang, Lin Shi, Kang-Lai Wei, Jiang-Hui Zeng, Jing-Jing Zeng, Gang Chen

**Affiliations:** ^1^ Department of Pathology, The First Affiliated Hospital of Guangxi Medical University, Nanning, China; ^2^ Department of Medical Oncology, The First Affiliated Hospital of Guangxi Medical University, Nanning, China; ^3^ Department of Pathology, Maternal and Child Health Hospital of Guangxi Zhuang Autonomous Region, Nanning, China; ^4^ Department of Radiotherapy, Guangxi Medical University Cancer Hospital, Nanning, China; ^5^ Department of Pathology, The Second Affiliated Hospital of Guangxi Medical University, Nanning, China; ^6^ Department of Clinical Laboratory, The Third Affiliated Hospital of Guangxi Medical University/Nanning Second People’s Hospital, Nanning, China

**Keywords:** molecular docking, dual-specificity protein phosphatase 1, immune landscape, cancer associated fibroblasts, ovarian carcinoma

## Abstract

**Introduction:** We aimed to explore the abnormal expression of dual-specificity protein phosphatase 1 (DUSP1) and its latent molecular mechanisms in ovarian carcinoma (OVCA).

**Materials and Methods:** Two clinical cohorts collected from two different hospitals were used to evaluate the expression of DUSP1 protein in OVCA tissues. RNA-sequencing and microarray datasets were utilised to verify DUSP1 expression at mRNA levels in both OVCA tissues and in the peripheral blood of OVCA patients. Furthermore, an integrated calculation was performed to pool the standard mean difference (SMD) from each cohort in order to comprehensively assess the expression of DUSP1 in OVCA. Furthermore, we examined the relationship among DUSP1, tumour microenvironment (TME), and chemotherapy resistance in OVCA. Moreover, we used pathway enrichment analysis to explore the underlying mechanisms of DUSP1 in OVCA.

**Results:** A pooled SMD of −1.19 (95% CI [−2.00, −0.38], *p* = 0.004) with 1,240 samples revealed that DUSP1 was downregulated in OVCA at both mRNA and protein levels. The area under the receiver operating characteristic curve of 0.9235 indicated the downregulated DUSP1 in peripheral blood may have a non-invasive diagnostic value in OVCA. Through six algorithms, we identified that DUSP1 may related to tumour-infiltrating T cells and cancer associated fibroblasts (CAFs) in OVCA. Pathway enrichment demonstrated that DUSP1 might participate in the mitogen-activated protein kinase (MAPK) signalling pathway. Furthermore, DUSP1 may have relations with chemotherapy resistance, and a favourable combining affinity was observed in the paclitaxel-DUSP1 docking model.

**Conclusion:** DUSP1 was downregulated in OVCA, and this decreasing trend may affect the infiltration of CAFs. Finally, DUSP1 may have a targeting relation with paclitaxel and participate in MAPK signaling pathways.

## Introduction

Malignant ovarian tumours threaten female health outcomes, and such tumours are considered to be the third-most common genital system cancer worldwide [1]. Ovarian cancer (OVCA) is a common type of gynaecological cancer that is well known for its highly mortality and recurrence rates [2–5]. In recent decades, treatment of OVCA has seemingly improved, but such treatment is still challenging due to the complex clinical manifestations, biological features and molecular mechanisms [6–11]. Interval debulking surgery (IDS) is used to reduce the number of tumour cells and is typically followed by neoadjuvant chemotherapy (NACT), including carboplatin and paclitaxel [12–16]. But OVCA patients often still meet with an unfavourable prognosis due to late diagnosis [17–21]. Further study of the mechanism of OVCA will be helpful to find effective measures for early diagnosis.

Dual-specificity protein phosphatase 1 (DUSP1), a member of dual-specificity phosphatases, is a threonine-tyrosine dual-specificity phosphatase that serves as a crucial factor in the inactivation of different mitogen-activated protein kinase (MAPK) isoforms [22, 23]. DUSP1 is also an autophagy modulator that plays a key role in the ferroptosis in a context-dependent manner [24]. The function of DUSP1 in the growth, migration, and invasion of OVCA cells has widely attracted the attention of researchers. It is reported that DUSP1 is a novel therapeutic target for meningioma patients and that the inhibition of DUSP1 suppresses the tumour growth *in vivo* [25]. DUSP1 downregulates the expression of the mesenchymal marker Snail by inactivating JNK and ERK, which impairs the migration and invasion of prostate cancer [26]. A related study reported that DUSP1 remarkably suppresses the invasion and metastasis of hepatocellular carcinoma (HCC) cells [27]. Moreover, DUSP1 might serve as a promising target for improving the effective use of the treatment Gemcitabine in pancreatic cancer patients [28]. DUSP1 has garnered significant attention from gynaecologists because of its effective performance in the treatment of other cancers. Another study has shown that DUSP1 regulates the expression of P-glycoprotein in OVCA cells to induce paclitaxel resistance [29]. Researchers also found that the expression of DUSP1 is downregulated in ovarian cancer stem cells [30] and that DUSP1 is regarded as a prognostic seed gene in OVCA [31]. In addition, DUSP1 improves the development of endometrioid adenocarcinoma by activating the mitogen-activated protein kinase/extracellular signal-regulated kinase pathway [32].

Although there has been some research done regarding the function of DUSP1 in OVCA, the mechanism of DUSP1 in OVCA still unknown. In the present study, we examined the expression status of DUSP1 protein within clinical OVCA cases via immunohistochemistry (IHC) staining; we then verified the expression level of DUSP1 mRNA in OVCA via high-throughput datasets. Furthermore, the downregulated trend of DUSP1 was verified via the peripheral blood of OVCA patients. The role of DUSP1 in the tumour microenvironment (TME) of OVCA was explored *via* estimating immune infiltration. *Via* the Kyoto Encyclopedia of Genes and Genomes (KEGG), Reactome Pathway Database, and gene ontology (GO) enrichment analysis, we studied the underlying molecular mechanisms of DUSP1 in OVCA. Moreover, we explored the relationship among drug susceptibility. DUSP1 and molecular docking revealed there was a favourable affinity between DUSP1 and paclitaxel. All of these steps aimed to explore the role of DUSP1 and its underlying molecular mechanisms in OVCA.

## Materials and Methods

### The Expression Status of Dual-Specificity Protein Phosphatase 1 Protein in Clinical Samples of Ovarian Cancer

OVCA and corresponding non-tumour ovarian tissues were collected from the First Affiliated Hospital of Guangxi Medical University (IHC cohort-1, *n* of OVCA = 60, *n* of non-tumour = 30) and the Second Affiliated Hospital of Guangxi Medical University (IHC cohort-2, *n* of OVCA = 15, *n* of non-tumour = 15), China. Our study has been approved by the Ethics Committee of the First Affiliated Hospital of Guangxi Medical University (2020-KY-E-095) and the Ethics Committee of the Second Affiliated Hospital of Guangxi Medical University (2021-KY-0130). All patients signed the informed consent. Table 1 demonstrated the clinicopathological parameters of the patients in our study.

**TABLE 1:** Basic information of included patients with ovarian carcinoma in this study.

Clinicopathological parameters		*n* of cases
Histological subtype	serous cystadenocarcinoma	61
	mucinous cystadenocarcinoma	13
	Mixed	1
Pathological grade	G1	15
	G2	21
	G3	23
	Unknown	16
Age	<50	32
	≥50	43
Clinical stage	I	1
	II	8
	III	31
	IV	6
	Unknown	15
T stage	T1	15
	T2	8
	T3	37
	Unknown	15
Lymph node metastasis	N0	53
	N1	7
	Unknown	15
Metastasis	M0	54
	M1	6
	Unknown	15
Human epididymis protein 4 (HE4)	Negative	26
	Positive	34
	Unknown	15

To determine the expression of DUSP1 at the protein level, we conducted IHC staining experiments with tissue samples, DUSP1 antibody (Biorbyt, orb216089, rabbit-anti human). All operations were conducted observing the manufacturer’s instruction. Formalin-fixed and paraffin-embedded tissue slides were soaked in xylene for deparaffinization. Anhydrous ethanol, 95% ethanol and 75% ethanol were used for rehydration. Ethylenediaminetetraacetic acid buffer (1:50, pH = 8.0) was carried out to retrieve antigen. After PBS soak, inactivation of endogenous peroxidase was accomplished via 3% H_2_O_2_ at 20°C for 10 min. The rabbit-anti human DUSP1 polyclonal antibody (dilution 1:200) was incubated at 37°C for 70 min. Universal mouse/rabbit secondary antibody was added into the tissue slides and placed in room temperature for 20 min, followed by PBS soak. Horseradish peroxidase-conjugated goat-anti rabbit IgG was added into the tissue slides and placed in 25°C for 20 min, followed by PBS soak. Diaminobenzidine was used to colorate and hematoxylin was used to counterstain. 75%, 85%, 95%, and 100% ethanol were carried out for dehydration and neutral gum was used to seal tissue slides. Simultaneously, we stained the tissue slides without the primary antibody (the rabbit-anti human DUSP1 polyclonal antibody) as negative controls (Supplementary Figure S1). We also prepared the positive controls following the manufacturer’s instruction, which reads prostate carcinoma tissues as positive controls (Supplementary Figure S2).

Two pathologists evaluated the scores of staining with microscopes independently. The staining intensity with no, light, moderate and strong staining gained 0, 1, 2, 3 point respectively. The scores of positive cells in visual field followed the criteria: 0%–5% (0 point), 6%–25% (1 point), 26%–50% (2 point), 51%–75% (3 point) and >75% (4 point). Finally, we calculated the IHC staining score via multiplying the intensity score and positive cells score [33, 34].

### Screening OVCA-Associated High-Throughput Datasets

In order to assess the expression status of DUSP1 mRNA in ovarian carcinoma, we searched the Cancer Genome Atlas (TCGA), the Genotype-Tissue Expression (GTEx), and the International Cancer Genome Consortium (ICGC) databases for tertiary RNA-sequencing (RNA-seq) datasets of OVCA and normal ovaries. We also retrieved ArrayExpress, Sequence Read Archive (SRA), Gene Expression Omnibus (GEO), and Oncomine databases to collect microarrays. Datasets meeting the following criteria were excluded: 1) OVCA or non-tumour samples less than three; 2) cases not from *Homo sapiens*; 3) patient cases or cell lines that contained drugs, siRNA, or radiation; and 4) probes not matching official gene symbols. For the raw data from the microarrays, an affy package of R was ultilised to combine the raw data and conduct a robust multi-array average (RMA) algorithm [35]. Then we integrated databases from same platforms and removed the batch effects *via* sva package [36]. The data retrieval flow diagram is shown in Supplementary Figure S3. As of July 30, 2021, 16 datasets were included (Table 2).

**TABLE 2:** General characteristics of microarray and RNA-sequencing datasets on ovarian carcinoma.

Study	Test method/Platform	Country	Year	OVCA group	Non-tumor ovary controls
GSE26712	GPL96	United States	2011	185	10
GSE6008	GPL96	United States	2007	99	4
GSE105437	GPL570	South Korea	2017	10	5
GSE29450	GPL570	United States	2011	10	10
GSE18520	GPL570	United States	2009	53	10
GSE10971	GPL570	Canada	2008	13	24
GSE54388	GPL570	United States	2017	16	6
GSE14407	GPL570	United States	2009	12	12
GSE36668	GPL570	Norway	2012	4	4
GSE119054	GPL19615	China	2019	6	3
GSE66957	GPL15048	United States	2015	57	12
GSE146553	GPL6244	United States	2020	46	9
GSE124766	GPL6480	Germany	2020	20	8
GSE132289	GPL20301	United Kingdom	2020	5	3
GSE155310	GPL18573	United Kingdom	2020	21	6
TCGA_GTEx_OVCA	RNA-seq	United States	2021	379	88

OVCA, ovarian carcinoma.

### Statistical Analysis of Dual-Specificity Protein Phosphatase 1 Expression in Ovarian Carcinoma Tissues

We used the Student’s *t*-test to compare the expression level of DUSP1 between OVCA and non-tumour tissues via GraphPad Prism 8 software (CA, United States). A two-tailed *p* value ≤0.05 indicated the differences were statistically significant. Receiver operating characteristic (ROC) curves were drawn to evaluate the discriminatory capacity of DUSP1 between OVCA and non-tumour samples. An area under curve (AUC) over 0.7 indicates a moderate discriminatory ability. We then performed an integrated study to comprehensively evaluate the expression status of DUSP1 in OVCA by pooling the data from the microarrays, RNA-seq, and in-house IHC. Stata v15.1 software (TX, United States) was utilised to calculate the standard mean difference (SMD) and a draw summary ROC (sROC) curve. The heterogeneity among the groups was assessed by a chi-square-based *Q*-test and *I* ^2^ statistical analysis. *I* ^2^ ≥ 50% and *p* value of *Q*-test ≤ 0.05 indicates existent heterogeneity and that a random effect model should be utilised to calculate pooled SMD; otherwise, a fixed effect model should be chosen. Egger’s test was used to assess the publication bias, and a *p* value ≥0.05 indicated no publication bias [14, 37, 38].

### Relations Between Dual-Specificity Protein Phosphatase 1 Expression and Tumour Microenvironment Landscape in Ovarian Carcinoma

For estimating the TME composition, an Immunedeconv R package was used to evaluate immune infiltration. This package integrates six algorithms: EPIC, MCP-counter, xCell, quanTIseq, TIMER, and CIBERSORT [39]. The TME landscape was constructed using a combination of ggplot2 and a pheatmap package. Meanwhile, we conducted Pearson’s correlation analysis to explore the relations between DUSP1 expression and cancer associated fibroblasts (CAFs) infiltration in OVCA.

### Pathway Analysis and Protein–Protein Interaction Network Construction

To make the underlying mechanisms of DUSP1 in OVCA clearer, we conducted pathway analysis. First, we calculated the Pearson’s correlation coefficient between the expression of DUSP1 and other genes in our included datasets, and the genes whose absolute value of correlation coefficient ≥0.4 were obtained as initial correlated expressed genes (CEGs) of DUSP1. The genes that appeared in at least three datasets were reckoned as ultimate correlated genes of DUSP1. Moreover, we calculated the SMD of every gene with a meta package of R and regarded the genes with *p* < 0.05 as differently expressed genes (DEGs) in OVCA. We then took the intersection genes of CEGs and DEGs to perform enrichment analysis [34].

We used the R package clusterProfiler to conduct GO term annotation and KEGG pathway enrichment analysis [40]. Furthermore, Reactome pathway enrichment was accomplished via the online tools KOBAS 3.0 [41].

### Prediction of Chemotherapeutic Response for Ovarian Carcinoma Patients

To further explore the clinical significance of DUSP1, we predicted the chemotherapeutic response for each sample in the TCGA-OVCA cohort based on the Genomics of Drug Sensitivity in Cancer (GDSC) database. The prediction was implemented by a pRRophetic R package, which estimated the half-maximal inhibitory concentration (IC50) of every sample via ridge regression [42]. The Wilcoxon test was used to compare the IC50 of OVCA patients between the high- and low-DUSP1 expression groups.

### Molecular Docking

To explore the targeting relationship between DUSP1 proteins and chemotherapeutic drugs, we performed molecular docking. First, we searched the crystal structure of DUSP1 proteins using the RCSB Protein Data Bank (RCSB PDB) database and downloaded a .pdb* file. Then, PyMOL software was ultilised to remove the nonspecific chains and solvent molecules. The 2D structures of the drugs were identified using the PubChem database, and we simultaneously used ChemBio3D software to minimise energy by an MM2 force-field-steepest descent algorithm; we then saved the structures as .mol2* files. Moreover, AutoDockTools 4 software was utilised to prepare the protein receptors and ligands according to the following steps: 1) add hydrogens to protein receptors; 2) compute and generate charges and save protein structures as .pdbqt* files; 3) generate the charges on the ligand atoms with Gasteiger models; and 4) adjust active torsions and save ligands as .pdbqt* files.

Next, non-flexible docking was conducted in AutoDock Vina, which established docking models via the Lamarckian genetic algorithm [43]. AutoDock Vina predicts the binding affinity (kcal/mol) of every conformation and outputs the result as a .pdbqt* file. Each conformation was assessed by simulating the docking of affinity-free energy between the protein receptors and ligands. Finally, we reckoned docking models with affinity energy ≤ -7 kcal/mol as ideal docking models and visualised the model with the lowest affinity energy via PyMOL.

## Results

### Expression Status of Dual-Specificity Protein Phosphatase 1 in Ovarian Carcinoma

**FIGURE 1:**
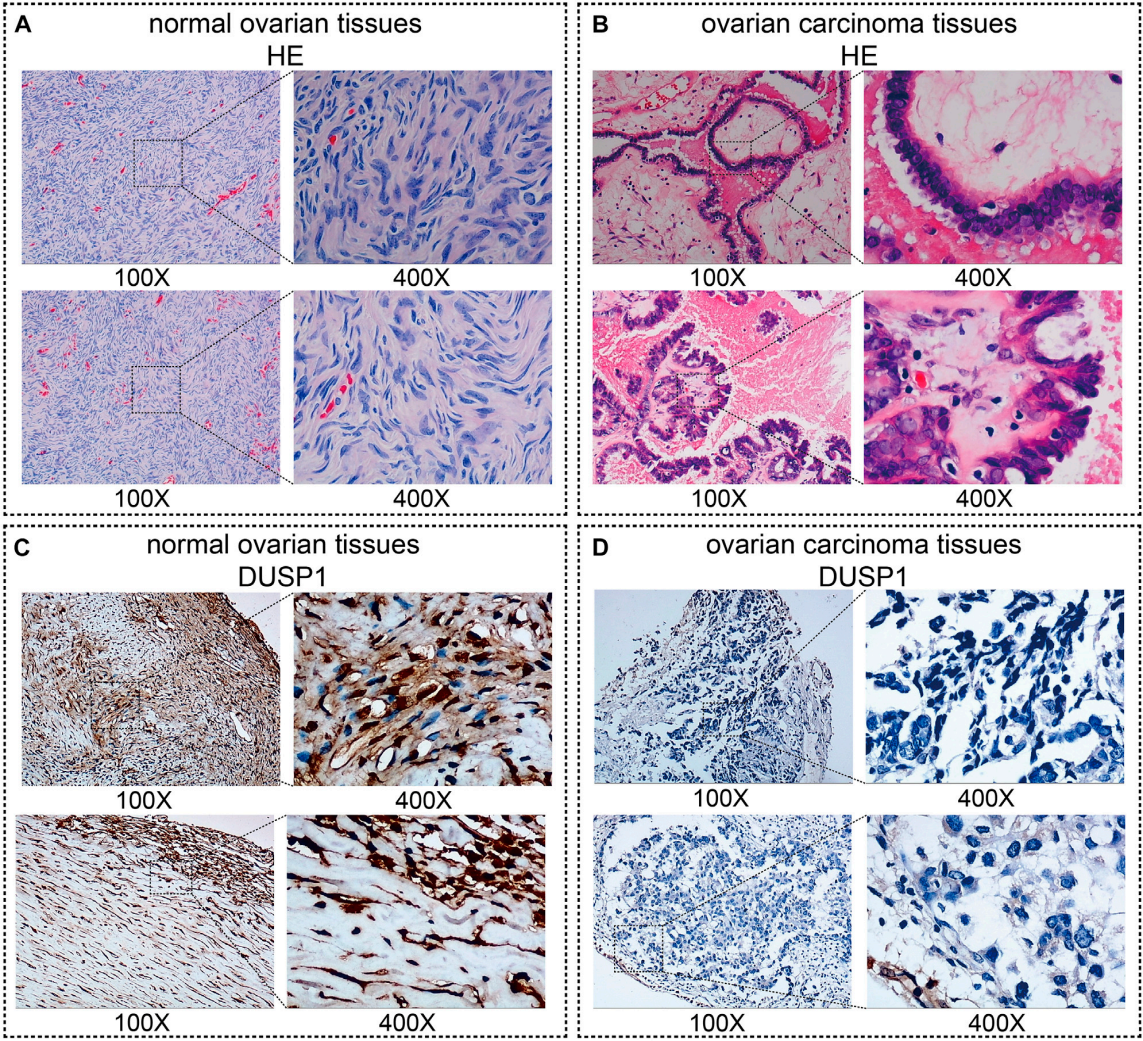
Images of normal ovarian and ovarian carcinoma tissues stained with hematoxylin-eosin (HE, **(A,B)**) and DUSP1 **(C,D)**.

**FIGURE 2:**
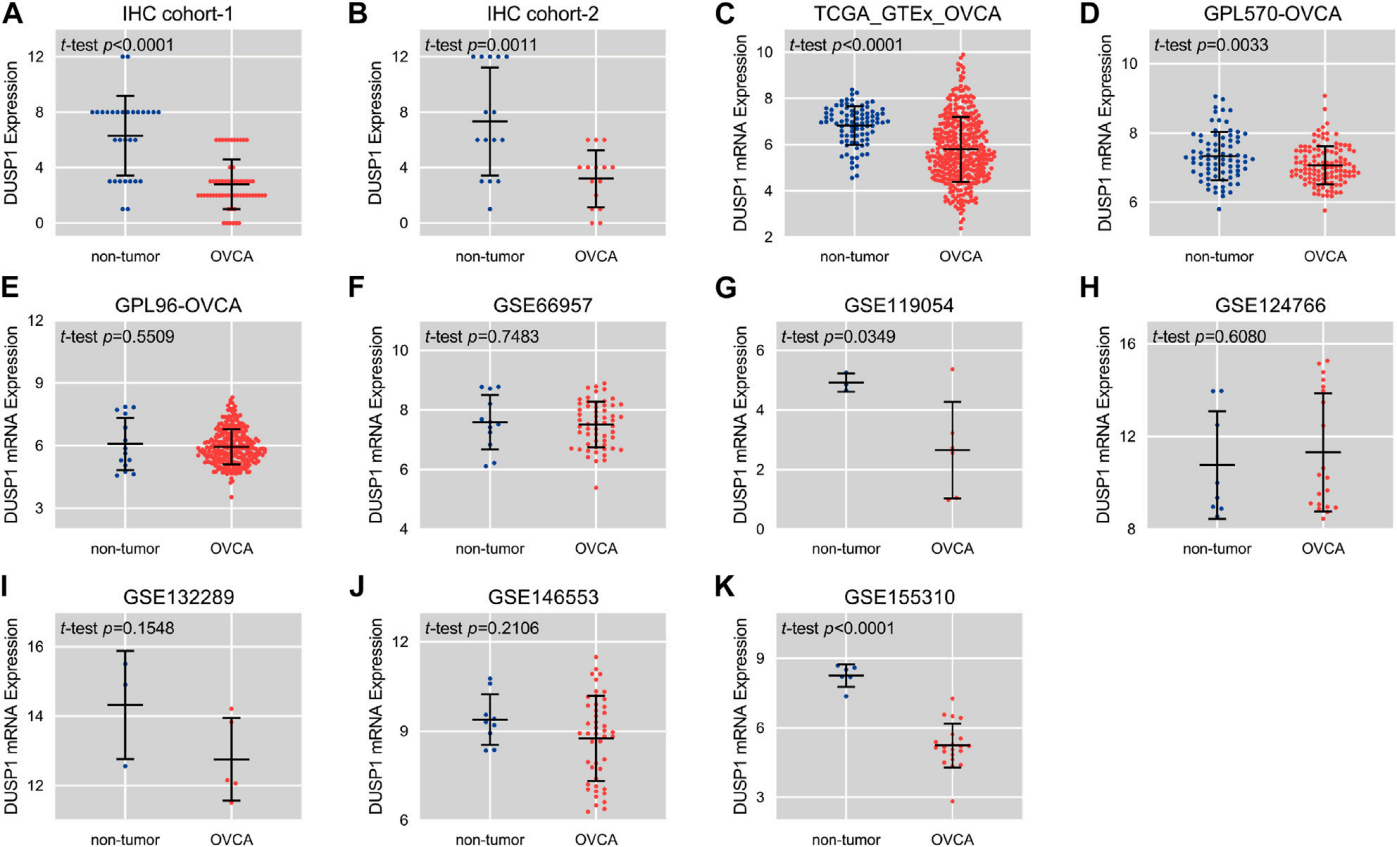
Scatter plots of the expression level of DUSP1 protein **(A,B)** and mRNA **(C–K)** in ovarian carcinoma (OVCA) and corresponding non-tumor tissues.

Figures 1A,B demonstrated the hematoxylin-eosin (HE) staining of normal ovarian and ovarian carcinoma tissues. Under microscope, IHC staining illustrated that no high expressions of DUSP1 protein were found in OVCA tissues but rather in non-tumour tissues (Figures 1C,D); the difference was considered statistically significant (IHC cohort-1, *p* < 0.0001; IHC cohort-2, *p* = 0.0011; Figures 2A,B). Considering the multiple subtype of OVCA, we compared the expression of DUSP1 in different subtypes and pathological grades. However, no significant differences were found between the serous cystadenocarcinoma and mucinous cystadenocarcinoma, nor among the different grades and stages (Supplementary Figure S4).

### Expression Status of Dual-Specificity Protein Phosphatase 1 mRNA in Ovarian Carcinoma

**FIGURE 3:**
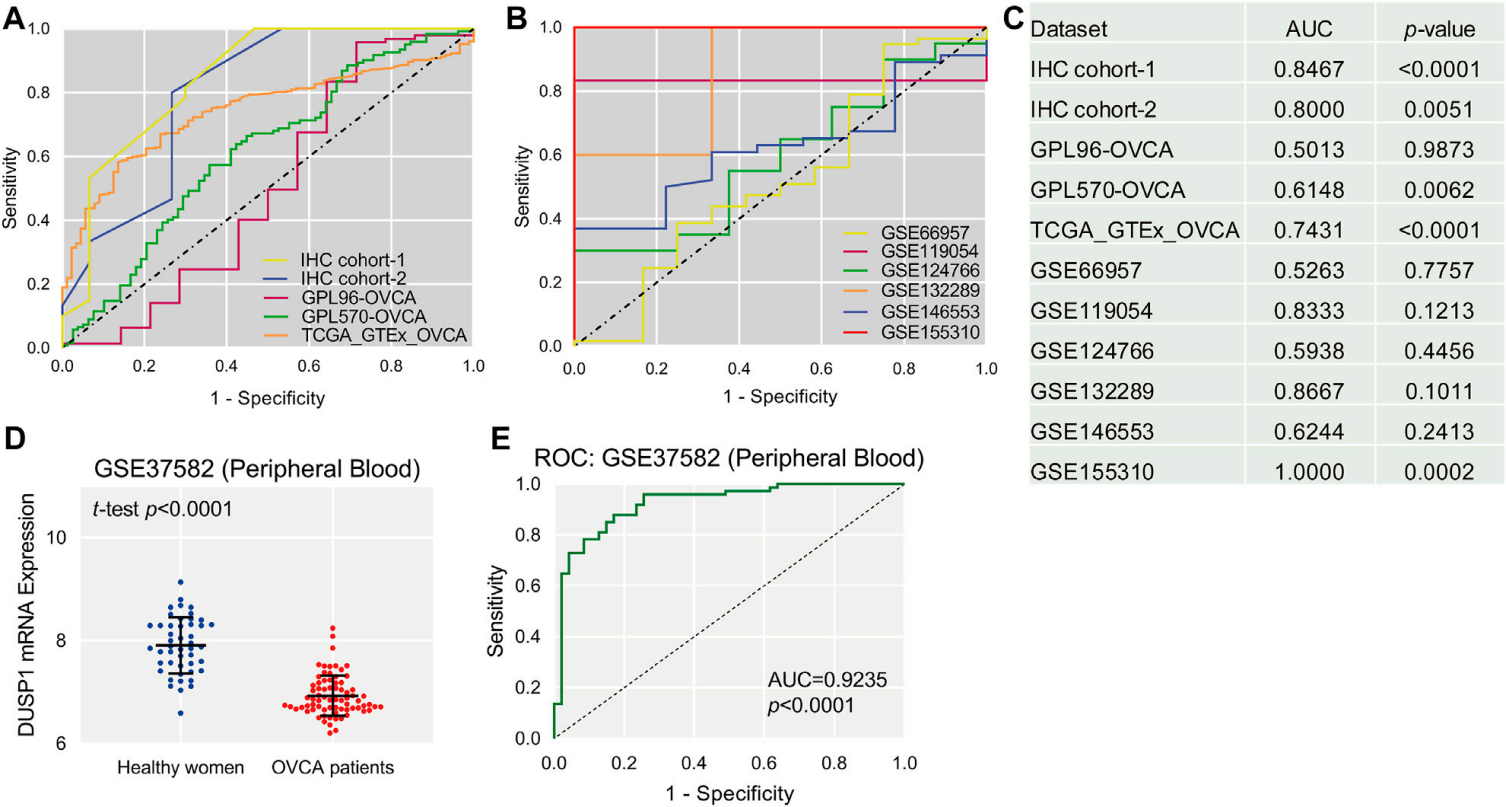
The receiver operating characteristic (ROC, **(A,B)**) curves of DUSP1 in ovarian carcinoma and area under curves (AUC, **(C)**). Scatter plot of DUSP1 mRNA expression in peripheral blood of ovarian carcinoma (OVCA) patients and healthy women **(D)** and ROC curve **(E)**.

Scatter plots showed the differences in DUSP1 mRNA expression levels between non-tumour tissues and OVCA tissues in every cohort (Figures 2C–K). Among the nine cohorts, four cohorts showed that the expression level of DUSP1 in OVCA was higher than that in non-tumour tissues, and the difference was statistically significant (TCGA_GTEx_OVCA, *p* < 0.0001; GPL570-OVCA, *p* = 0.0033; GSE11954, *p* = 0.0349; GSE155310, *p* < 0.0001). Furthermore, five other cohorts revealed no statistically significant difference in DUSP1 mRNA between OVCA and non-tumour tissues (GPL96-OVCA, *p* = 0.5509; GSE66957, *p* = 0.7483; GSE124766, *p* = 0.6080; GSE132289, *p* = 0.1548; GSE146553, *p* = 0.2106). The ROC curves for the above cohorts and the AUC are shown in Figures 3A–C.

After observing the favourable discriminatory ability of DUSP1 in OVCA tissue, we evaluated the expression level and diagnostic value of DUSP1 in bodily fluids of OVCA patients. From a microarray consisting of peripheral blood lymphoblastoid cells from 74 OVCA patients and 47 healthy women, GSE37582 was obtained and was utilised to assess the expression status of DUSP1 mRNA. The expression level of DUSP1 mRNA in the peripheral blood of OVCA patients was obviously lower than that of healthy women (*p* < 0.0001, Figure 3D), and the area under the ROC of 0.9235 indicated a favorable diagnostic capacity of downregulated DUSP1 mRNA in peripheral blood (*p* < 0.0001, Figure 3E).

### Comprehensive Analysis of Dual-Specificity Protein Phosphatase 1 Expression in Ovarian Carcinoma

**FIGURE 4:**
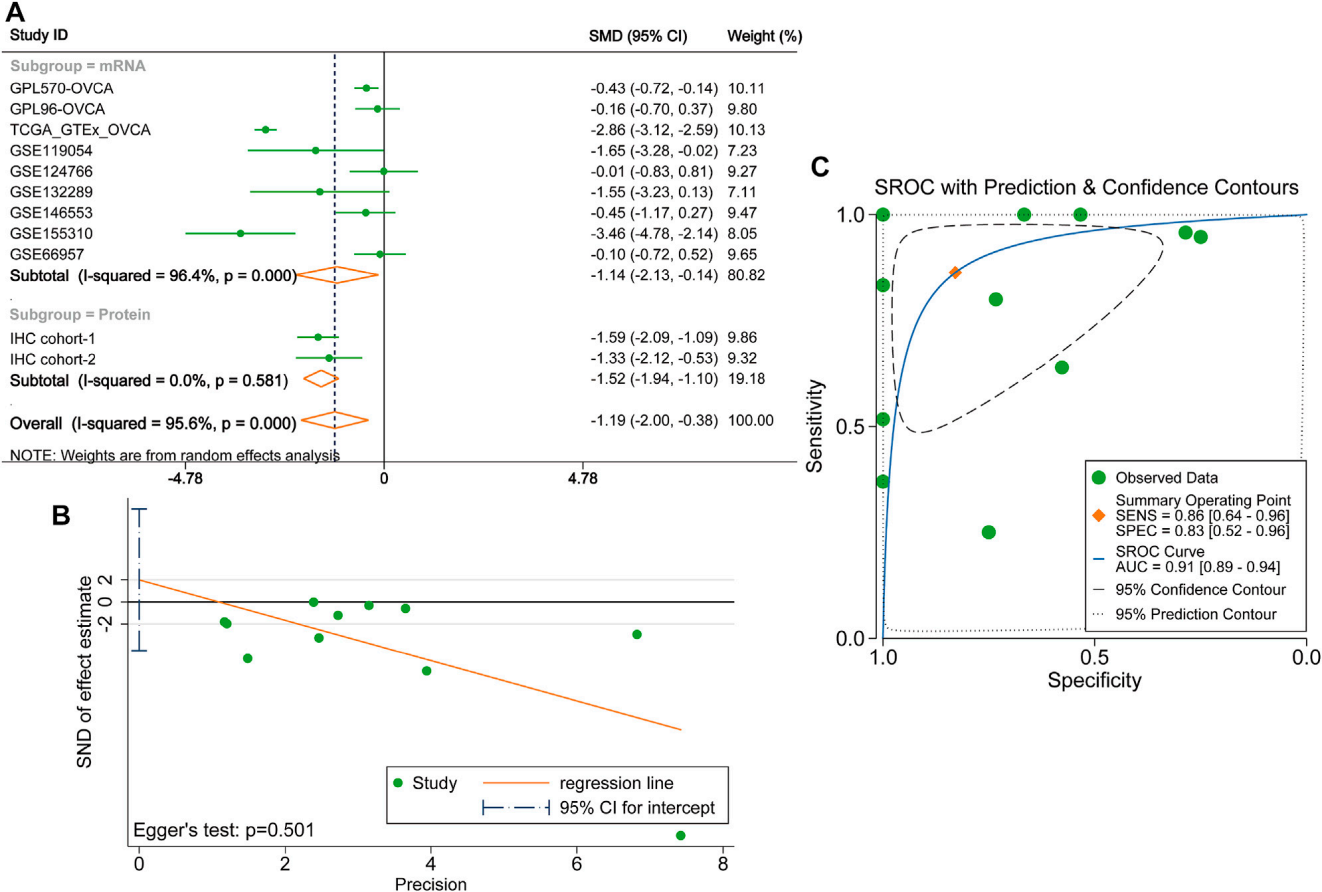
Integrated analysis on the expression of DUSP1 in ovarian carcinoma tissues. **(A)**Forest plot of combing standard mean difference of DUSP1 expression in ovarian carcinoma and normal ovary tissues. IHC, Immunohistochemical staining, two cohorts were from two different hospitals. OVCA, ovarian carcinoma. **(B)** Egger’s test for publication bias. **(C)** Summary receiver operating characteristic (sROC) curve of all the included cohorts.

Considering existing heterogeneity between studies (*I* ^2^ = 95.6%, *p* < 0.0001), the random effects model was used to calculate the pooled SMD of each cohort. The result of the subgroup analysis demonstrated that DUSP1 expression level was lower in OVCA tissues than that in non-tumour tissues (mRNA subgroup: SMD = −1.14, 95% CI [−2.13, −0.14], *p* = 0.025; protein subgroup: SMD = -1.52, 95% CI [−1.94, −1.10], *p* < 0.001). The integrated SMD was −1.19 (95% CI [−2.00, −0.38], *p* = 0.004, Figure 4A), indicating that the expression of DUSP1 is downregulated at both mRNA and protein levels in OVCA tissues. No publication bias was found by Egger’s test (*p* = 0.501, Figure 4B). The AUC of the sROC curve was 0.91 (95% CI [0.89, 0.94], Figure 4C).

### Relations Between Dual-Specificity Protein Phosphatase 1 and Tumour Microenvironment of Ovarian Carcinoma

**FIGURE 5:**
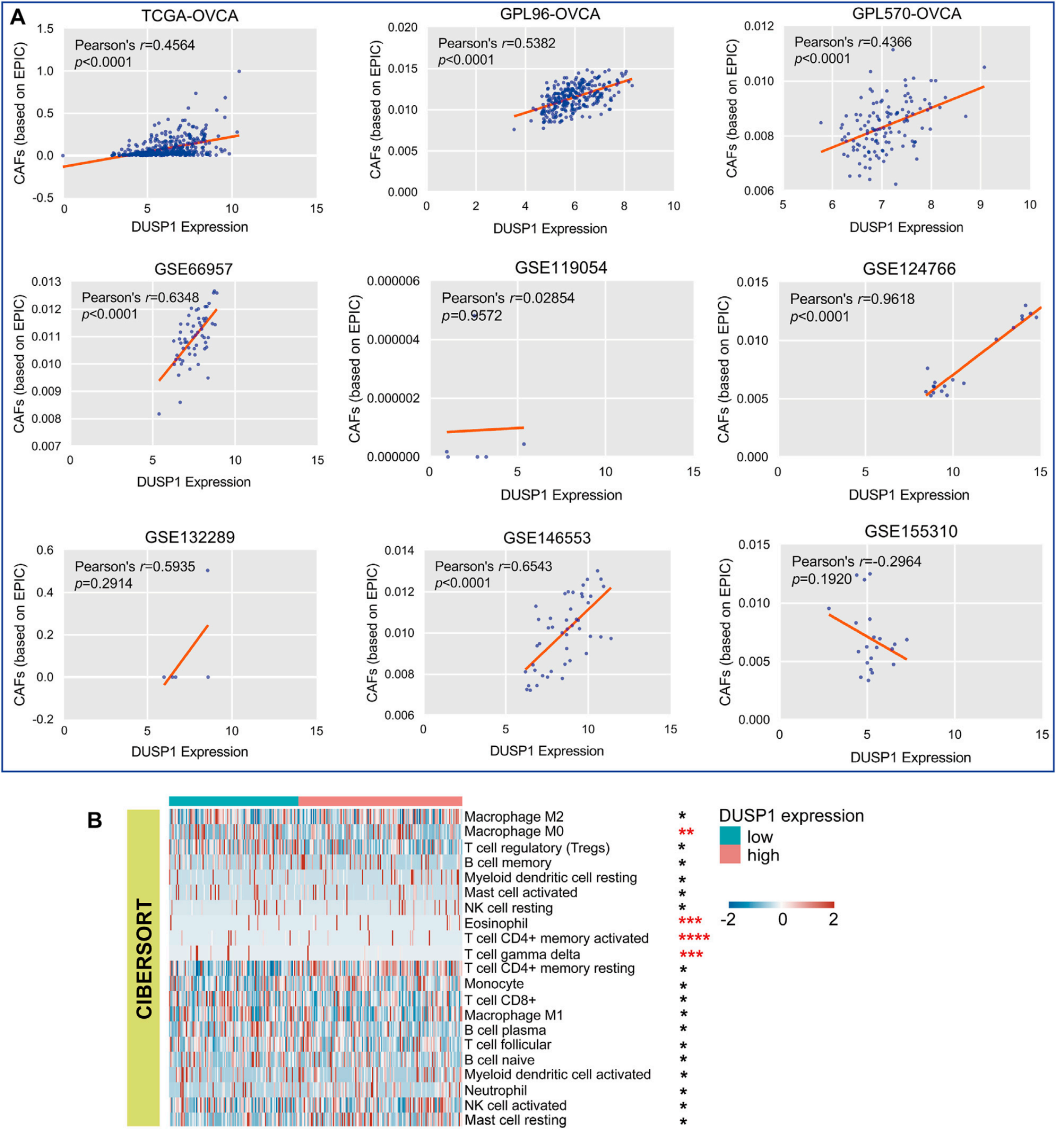
Relations of DUSP1 and tumor microenvironment in ovarian carcinoma. **(A)** Pearson’s correlation analysis on the DUSP1 expression and cancer associated fibroblasts (CAFs) infiltration in ovarian carcinoma tissues (based on EPIC algorithm. **(B)** A landscape of tumor microenvironment of ovarian carcinoma in low- and high-DUSP1 expression group based on CIBERSORT algorithms (****p ≤ 0.0001; ***p ≤ 0.001; **p < 0.05; *p ≥ 0.05).

As illustrated in Figure 5A, six cohorts showed that DUSP1 expression was positively correlated to the infiltrating CAFs in OVCA (Pearson’s *r* > 0.4, *p* < 0.0001). Among multiple types of cells in TME, Macrophage M0 and γδT cells presented less infiltration in the high-DUSP1 groups (Figure 5B).

### Enrichment Analysis

**FIGURE 6:**
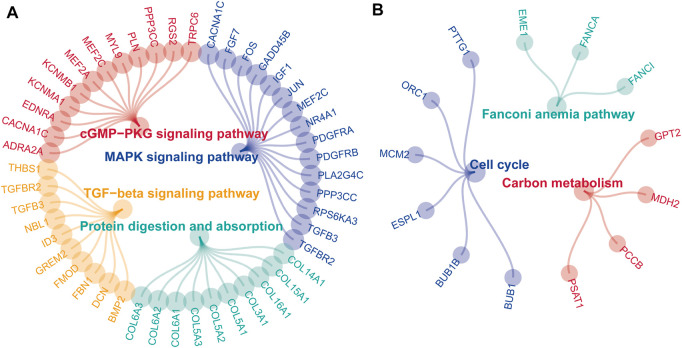
Enrichment plots of KEGG pathways of downregulated positive-correlated genes of DUSP1 **(A)** and upregulated negative-correlated genes of DUSP1 **(B)**.

**FIGURE 7:**
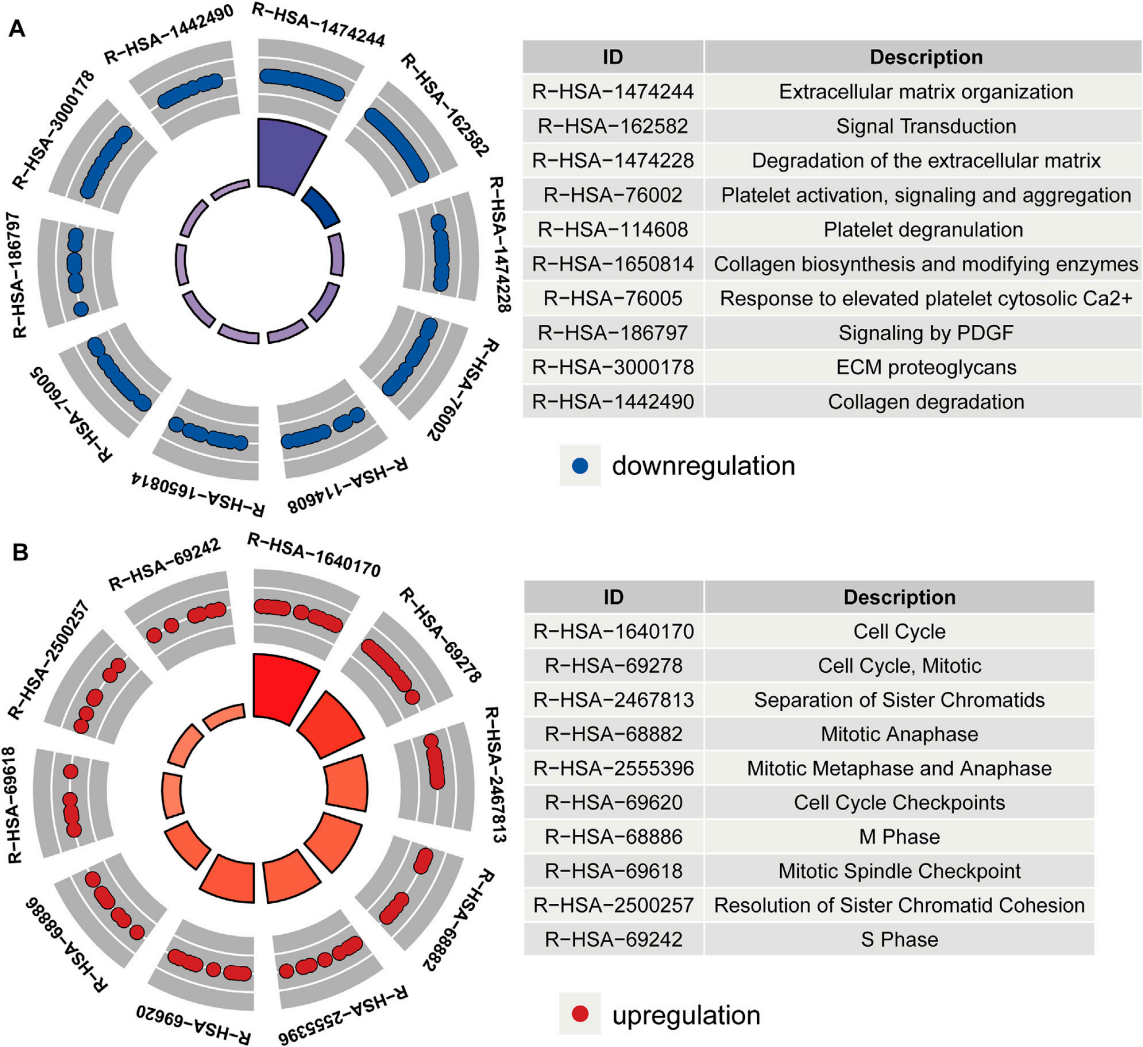
Circle plots of Reactome pathways enrichment of downregulated positive-correlated genes of DUSP1 **(A)** and upregulated negative-correlated genes of DUSP1 **(B)**.

**FIGURE 8:**
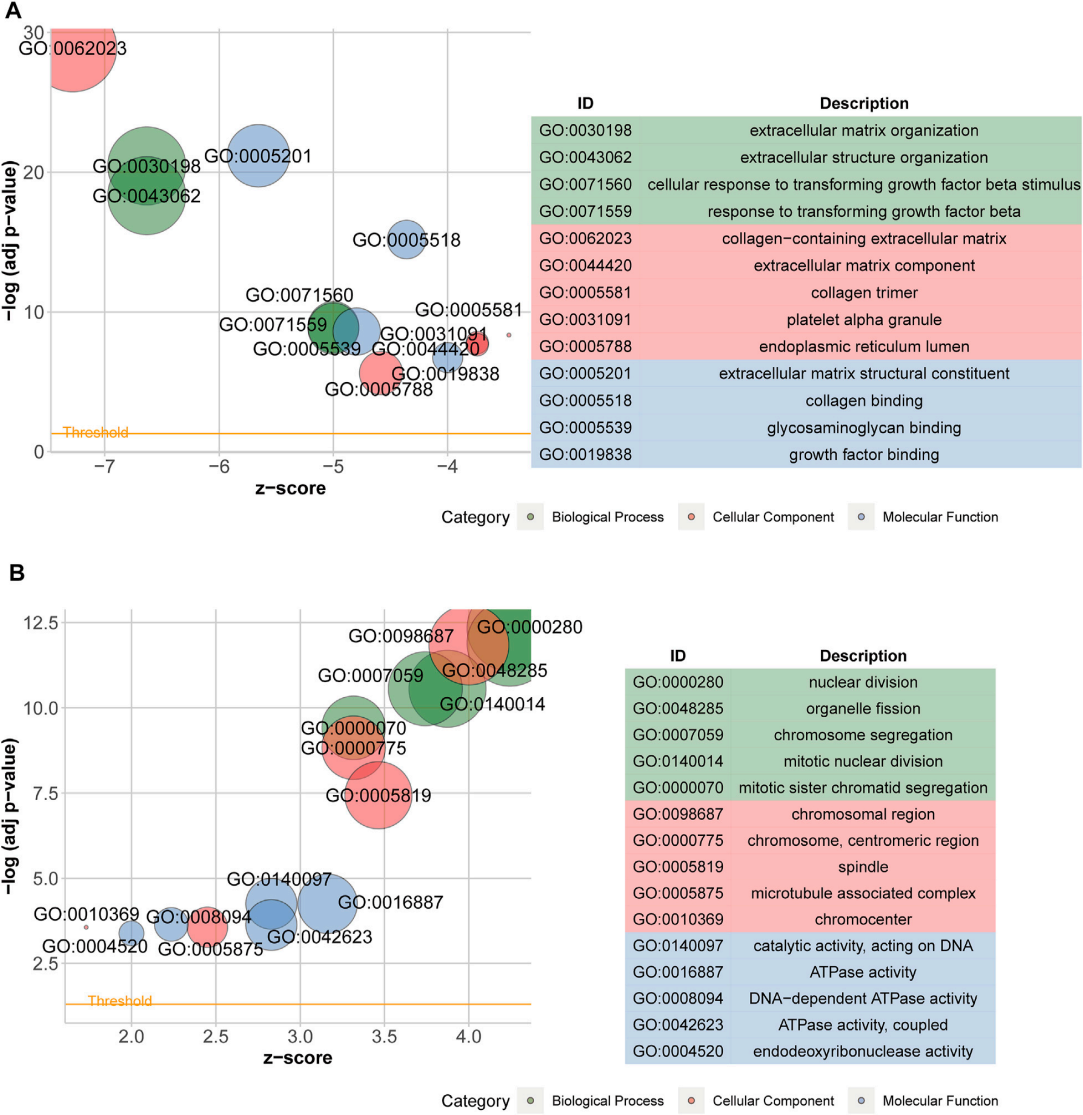
Bubble plots of GO enrichment analysis of downregulated positive-correlated genes of DUSP1 **(A)** and upregulated negative-correlated genes of DUSP1 **(B)**.

After acquiring 336 downregulated positive CEGs and 64 upregulated negative CEGs of DUSP1 in OVCA (Supplementary Figure S5), we performed enrichment analysis. As illustrated by crosstalk maps of KEGG pathways in Figure 6, downregulated positive CEGs of DUSP1 mostly participated in MAPK signalling pathways, cGMP-PKG signalling pathways, and TGF-β signalling pathways, etc., whereas a majority of upregulated negative CEGs of DUSP1 appeared in the pathways of cell cycles and oocyte meiosis (Supplementary Table S1). Similar results were also revealed via Reactome Pathway enrichment (Figure 7). GO enrichment revealed that downregulated CEGs of DUSP1 were enriched in terms related to extracellular matrix (GO: 0030198, GO: 0062023, GO: 0005201, etc.; Figure 8A); however, upregulated CEGs of DUSP1 were enriched in terms of cell cycle (GO: 0000280, GO: 0098687, GO: 0140097, etc.; Figure 8B).

### Role of Dual-Specificity Protein Phosphatase 1 in Chemotherapy Resistance and Molecular Docking

**FIGURE 9:**
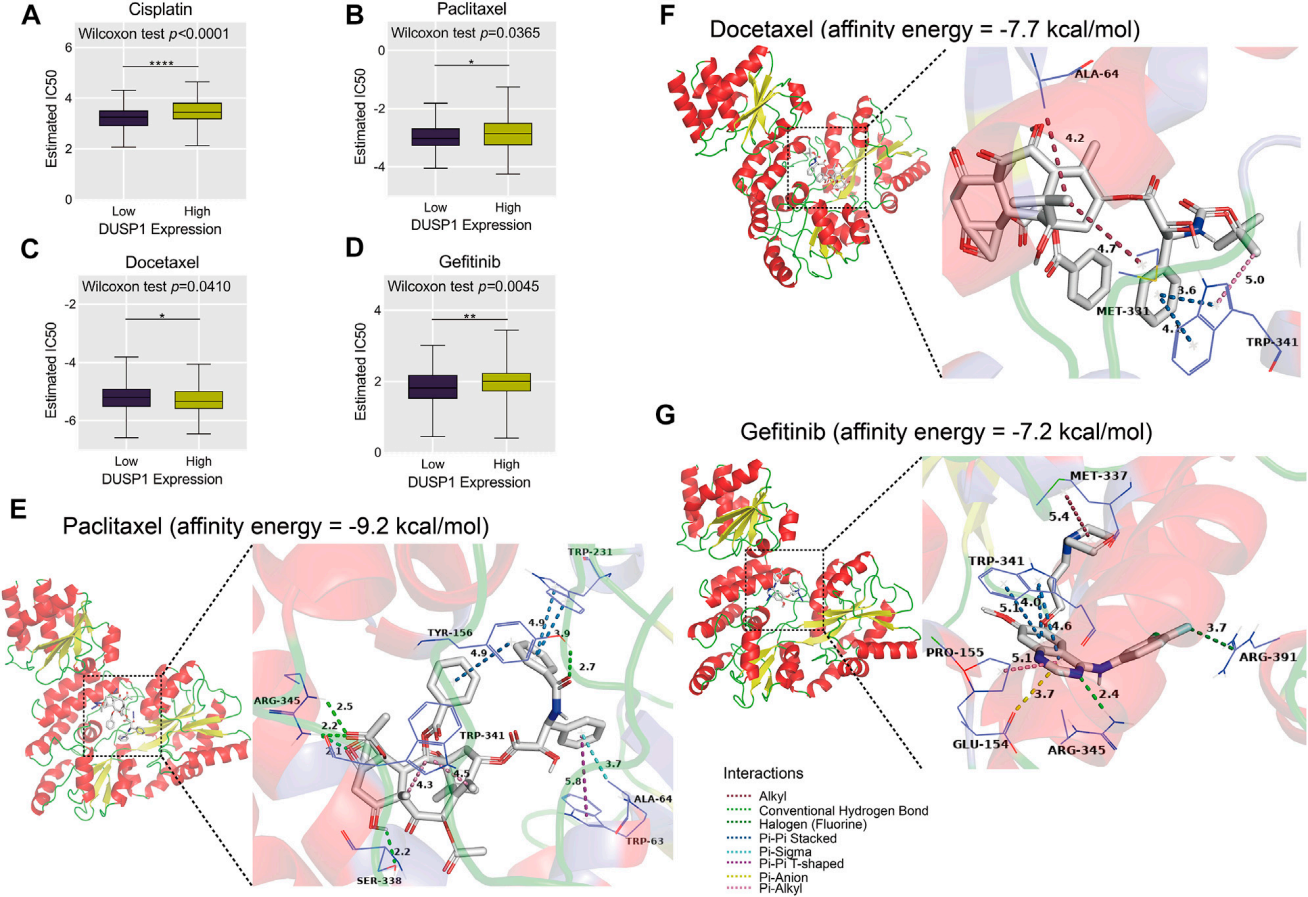
Estimated half-maximal inhibitory concentration (IC50) of cisplatin, paclitaxel, docetaxel and gefitinib in high- and low-DUSP1 group **(A–D)** and molecular docking models of drug-DUSP1 protein **(E–G)**.

Through ridge regression, we predicted the IC50 of cisplatin, paclitaxel, docetaxel, and gefitinib in the TCGA-OVCA cohort. The boxplots demonstrated that patients with high DUSP1 expression tended to have lower IC50 of cisplatin, paclitaxel, and gefitinib than patients with low *DUSP1* expression (Figures 9A,B,D); this indicated that OVCA patients with higher DUSP1 expression may respond better to cisplatin, paclitaxel, and gefitinib than patients with lower DUSP1 expression. By contrast, patients with high DUSP1 expression tended to have higher IC50 of docetaxel than patients with low DUSP1 expression (Figure 9C).

To further explore the interaction between the aforementioned drugs and DUSP1, molecular docking was conducted. A cisplatin-DUSP1 docking model was unsatisfactory (affinity energy = 0 kcal/mol), which meant that we did not display this docking model. The docking models paclitaxel-DUSP1 (affinity energy = −9.2 kcal/mol), docetaxel-DUSP1 (affinity energy = −7.7 kcal/mol), and gefitinib-DUSP1 (affinity energy = −7.2 kcal/mol) are included in Figures 9E–G.

## Discussion

In the present study, we demonstrated that the downregulated trend of DUSP1 protein in OVCA is based on clinical case analysis via IHC experiments (*n* of OVCA = 75, *n* of non-tumour = 45). Microarrays and RNA-seq cohorts also validated a decreasing trend of DUSP1 at mRNA levels in OVCA tissues via a large sample size (*n* of OVCA = 936, *n* of non-tumour = 214), which was also observed in peripheral blood of OVCA patients (*n* of OVCA = 74, *n* of healthy women = 47). Using multiple approaches, we simultaneously explored the relationship among DUSP1, TME, and the underlying molecular pathways in OVCA.

DUSP1 may play stimulative or suppressive roles in different types of tumours [23]. Some studies have shown the aberrant expression status of DUSP1 in malignant tumours such as prostate small cell carcinoma [44], liver cancer [45], and non-small cell lung cancer [46], which may affect the therapeutic effect of chemotherapeutic drugs. Interestingly, there has not yet been a study that has identified the dysregulation of DUSP1 at mRNA or protein levels in OVCA (based on PubMed database, as of September 3, 2021). In our study, we implemented a comprehensive analysis of the expression status of DUSP1 in OVCA and first revealed the downregulation of DUSP1 in OVCA tissues at both mRNA and protein levels through examining 1,240 samples via calculating and combining SMD. Furthermore, within our study, the downregulated DUSP1 was first demonstrated in the peripheral blood of OVCA patients among 121 samples.

The clinical impact of DUSP1 was absorbing. One previous study reported the prognostic value of DUSP1 in OVCA via high-throughput cohort [31]. However, no previous study investigated the distinguishing ability or diagnostic value of DUSP1 in OVCA. In the present study, we illustrated the sROC curve, and the AUC of 0.91 indicated that DUSP1 could commendably distinguish OVCA tissues from non-tumour tissues. The ROC curve of the peripheral blood from OVCA patients also presented a compelling discriminatory capacity and favourable diagnostic value of DUSP1 mRNA (AUC = 0.9235), which has never been reported before. DUSP1 mRNA in peripheral blood might be a diagnostic biomarker of OVCA, which still need further investigation and validation.

Previously, some studies have revealed that TME changes in ovarian cancer were relevant to clinical outcomes in patients and could serve as the basis of potential therapeutic approaches [47, 48]. Appropriate immune-targeted therapy is expected to improve the prognosis of ovarian cancer patients [49, 50]. CAFs were reported to accelerate the development of malignant tumors, including OVCA [51, 52]. CAFs could excrete extracellular matrix and participate in the remodeling of extracellular matrix, which resulted in the dynamic changes of TME [53]. Some studies revealed that CAFs could impact on multiple process in TME, such as angiogenesis, invasion, migration and immunosuppression [54]. However, the roles of CAFs were heterogeneous [55]. Some subtypes of CAFs would not facilitate the invasion, proliferation and metastasis of tumor cells, which played as a inhibitor in the development of tumors [56]. Tumour-infiltrating T cells (CD4^+^T cells and CD8^+^T cells, etc.) were identified to play crucial roles in OVCA [57]. The γδT cell is a part of the innate immune system, and previous studies identified this type of T cell as potentially able to discern the diverse antigens on the membranes of tumour cells and kill them with multiple innate cytotoxicity receptors [58–60]. In our study, we constructed a TME landscape of OVCA and demonstrated that the proportion of T cells was lower in the high*-*DUSP1 expression group compared to the low*-*DUSP1 expression group, whereas CAFs showed a converse trend, suggesting that DUSP1 may have a relationship with TME in OVCA and may be a target for immunotherapy.

DUSP1 is a member of the threonine/tyrosine DUSP family. DUSP1 can dephosphorylate threonine and tyrosine and acts as an inhibitor in p38/MAPK signalling [23]. One study reported that DUSP1 negatively regulated autophagy of OVCA cells via downregulating MAPK/ERKs signalling [61]. MAPK signalling pathways were demonstrated to regulate the proliferation, invasion, and epithelial-mesenchymal transition of OVCA cells [62, 63]. Activating the cGMP-PKG pathway could result in the activation of a MAPK signalling pathway and the proliferation and metastasis of cancer cells [64, 65]. Cell cycle pathways play a crucial role in the tumorigenesis and development of OVCA, and targeting the abnormal cell cycle might facilitate the treatment of OVCA [66–68]. In our work, we illustrated the correlated genes of DUSP1 in OVCA enriched in MAPK signalling pathway, cGMP-PKG signalling pathway and cell cycle pathway through pathway enrichment analysis. This kind of work may help us learn more about the molecular mechanisms of DUSP1 in OVCA.

Chemotherapy is a common treatment for OVCA patients [69]. DUSP1 was reported to have a relationship with resistance to paclitaxel, which is also commonly used to treat OVCA [29, 70]. Cisplatin and docetaxel are also used in chemotherapy to treat OVCA, and the resistance mechanisms and the way to enhance therapeutic effects have been widely studied [71–73]. Gefitinib is an EGFR-targeting compound and is identified to promote the prognosis of lung cancer [74, 75]. Interestingly, in our study, we illustrated that DUSP1 may be related to the resistance of cisplatin, paclitaxel, docetaxel, and gefitinib through predicting IC50. Furthermore, molecular docking revealed the interactions between compounds and DUSP1 proteins. For paclitaxel, there were conventional hydrogen bonds between paclitaxel molecules and ARG-345, between SER-338 and TYR-156 residues of DUSP1; there was pi-pi interaction and stacking between paclitaxel and TYR-156 and between TRP-231 and TRP-63 (T-shaped) residues, which jointly made this paclitaxel-DUSP1 docking model stable. Pi-stacking was also identified between docetaxel and TRP-341 and gefitinib and TRP-341, which indicated that docetaxel and gefitinib may have interactions with DUSP1 proteins.

Collectively, our study demonstrated the downregulation trend of DUSP1 in OVCA tissues at both the mRNA and protein levels; this was also verified in the peripheral blood of OVCA patients. In addition, DUSP1 may act as a tumour-suppressor in OVCA bye effecting TME and inhibiting MAPK signalling pathways. Moreover, DUSP1 and paclitaxel were proved to be a commpelling combination. However, it must be acknowledged that our study still has some limitations. First, there was a lack of clinical parameters; we were unable to fully elucidate the clinicopathological significance of DUSP1 in OVCA. In addition, the underlying pathways and transcriptional regulatory mechanisms of DUSP1 still require verification with experiments *in vivo* and *in vitro*.

## Conclusion

Briefly, via combining high-throughput datasets and clinical samples we illustrated that the expression of DUSP1 was lower in OVCA than that in normal ovarian tissues at both the mRNA and protein levels. This decreasing trend was also observed in the peripheral blood of OVCA patients, and it may have non-invasive diagnostic significance. The dysregulation of DUSP1 may be related to the resistance of chemotherapy, and it had a significant interaction with paclitaxel by means of molecular docking. Furthermore, we identified that DUSP1 may participate in MAPK signalling, cell cycle pathways and the regulation of CAFs.

## Data Availability

The datasets presented in this study can be found in online repositories. The names of the repository/repositories and accession number(s) can be found in the article/Supplementary Material.

## Supplementary Material

Supplementary Figure S1 Images of normal ovarian and ovarian carcinoma tissues stained with hematoxylin-eosin (HE) and negative control.

Supplementary Figure S2 Images of immunohistochemical staining of DUSP1 in prostate carcinoma tissues as positive controls.

Supplementary Figure S3 Flow chart of filtering ovarian carcinoma-related high-throughput datasets.

Supplementary Figure S4 Violin plots of DUSP1 expression in different histological subtypes **(A)**, pathological grades **(B)** and clinical stage **(C)** of ovarian carcinoma.

Supplementary Figure S5 Venn diagrams of intersection between differentially expressed genes in ovarian carcinoma and correlated expressed genes of DUSP1.
