# Supplementary material for: Downregulated Dual-Specificity Protein Phosphatase 1 in Ovarian Carcinoma: A Comprehensive Study With Multiple Methods
Source: Pathol Oncol Res. 2022 Jul 15;28:1610404. doi: 10.3389/pore.2022.1610404 (PMC9336223; doi:10.3389/pore.2022.1610404)
Supplement: Supplementary file 1 [file Table1.DOCX]

**Supplementary Table 1.** The KEGG pathway of *DUSP1*-correlated DEGs in OVCA tissues

| Gene | ID | Description | *p* value | adjusted *p* | Count |
| --- | --- | --- | --- | --- | --- |
| Positive correlated DEGs of *DUSP1* | hsa04350 | TGF-beta signaling pathway | <0.0001 | 0.002193 | 10 |
|  | hsa04974 | Protein digestion and absorption | <0.0001 | 0.002193 | 10 |
|  | hsa04510 | Focal adhesion | <0.0001 | 0.002193 | 14 |
|  | hsa04022 | cGMP-PKG signaling pathway | <0.0001 | 0.004647 | 12 |
|  | hsa04610 | Complement and coagulation cascades | 0.000198 | 0.009643 | 8 |
|  | hsa05031 | Amphetamine addiction | 0.000314 | 0.011761 | 7 |
|  | hsa05144 | Malaria | 0.00034 | 0.011761 | 6 |
|  | hsa04010 | MAPK signaling pathway | 0.000451 | 0.014056 | 15 |
|  | hsa04933 | AGE-RAGE signaling pathway in diabetic complications | 0.000601 | 0.016275 | 8 |
|  | hsa04068 | FoxO signaling pathway | 0.000839 | 0.020518 | 9 |
|  | hsa04270 | Vascular smooth muscle contraction | 0.000936 | 0.021926 | 9 |
|  | hsa05418 | Fluid shear stress and atherosclerosis | 0.001279 | 0.026041 | 9 |
|  | hsa04670 | Leukocyte transendothelial migration | 0.001422 | 0.026617 | 8 |
|  | hsa05218 | Melanoma | 0.002374 | 0.039587 | 6 |
|  | hsa05231 | Choline metabolism in cancer | 0.002544 | 0.039587 | 7 |
|  | hsa04921 | Oxytocin signaling pathway | 0.002597 | 0.039587 | 9 |
|  | hsa05214 | Glioma | 0.002921 | 0.041500 | 6 |
| Negative correlated DEGs of *DUSP1* | hsa04110 | Cell cycle | <0.0001 | 0.001027 | 6 |
|  | hsa01200 | Carbon metabolism | 0.001648 | 0.035216 | 4 |
|  | hsa03460 | Fanconi anemia pathway | 0.001709 | 0.035216 | 3 |

Note: DEGs, differently expressed genes
