# Supplementary figures and images for: Downregulated Dual-Specificity Protein Phosphatase 1 in Ovarian Carcinoma: A Comprehensive Study With Multiple Methods
Source: Pathol Oncol Res. 2022 Jul 15;28:1610404. doi: 10.3389/pore.2022.1610404 (PMC9336223; doi:10.3389/pore.2022.1610404)

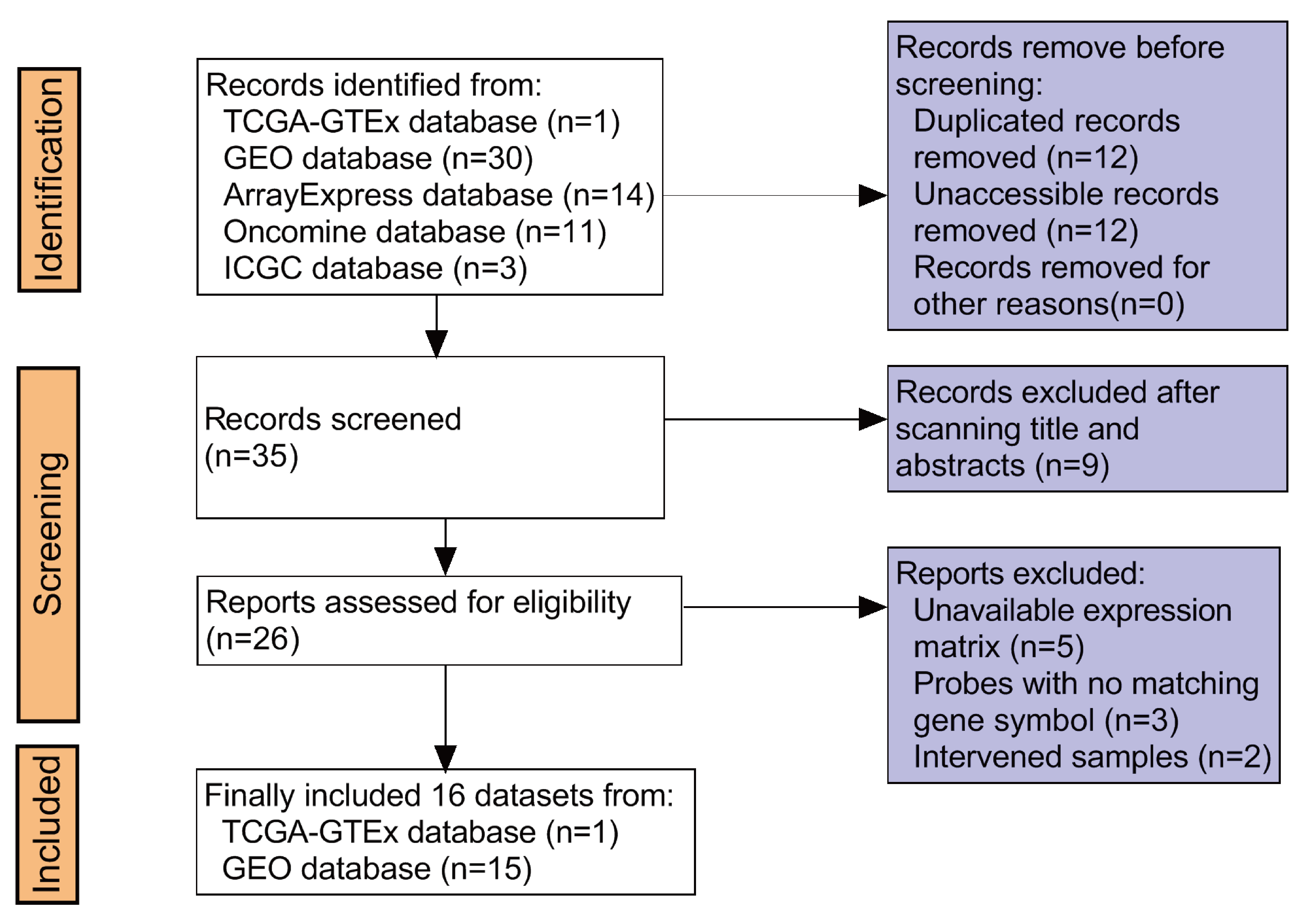

Supplement: Supplementary file 2 [file Image3.TIF]

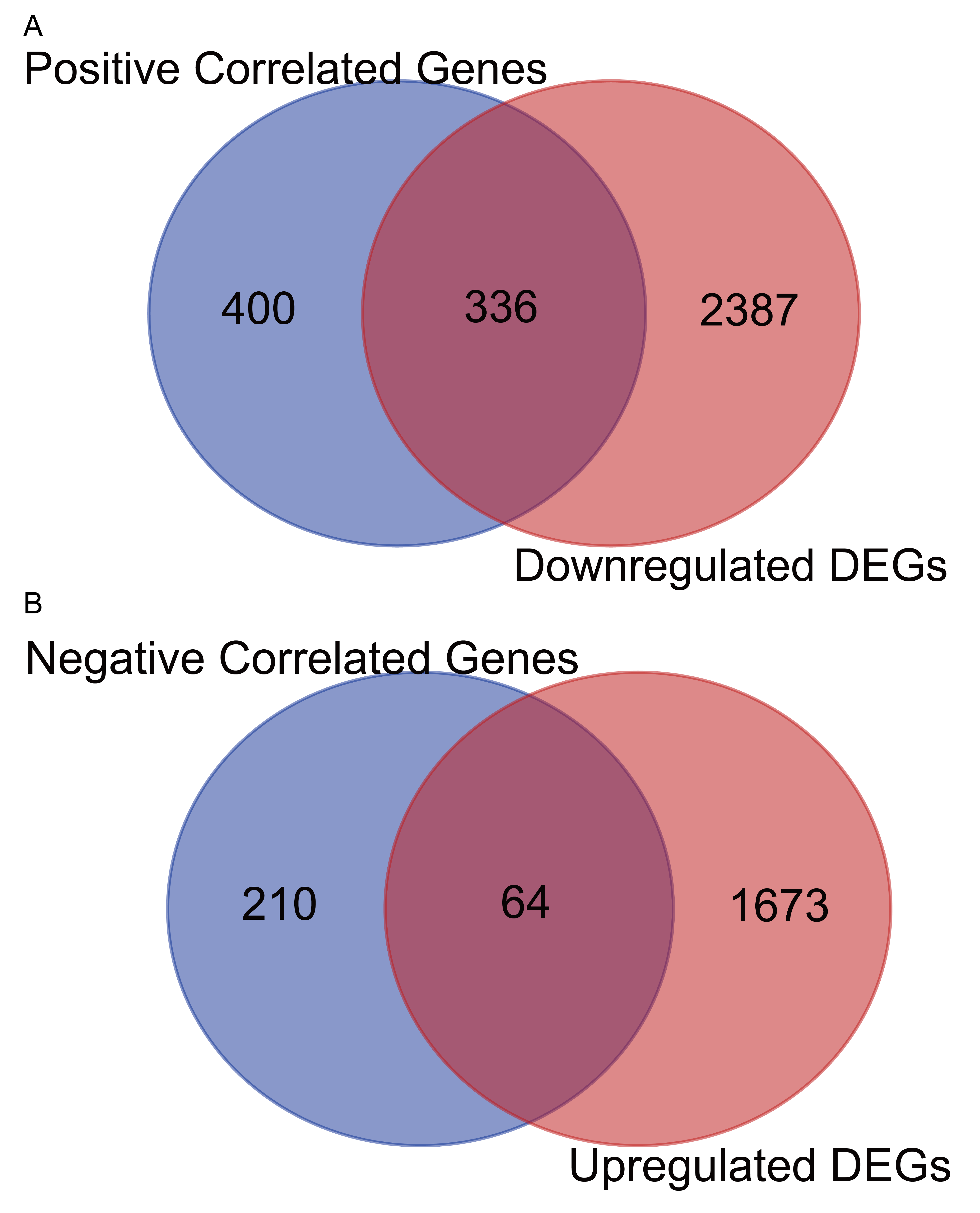

Supplement: Supplementary file 3 [file Image5.PNG]

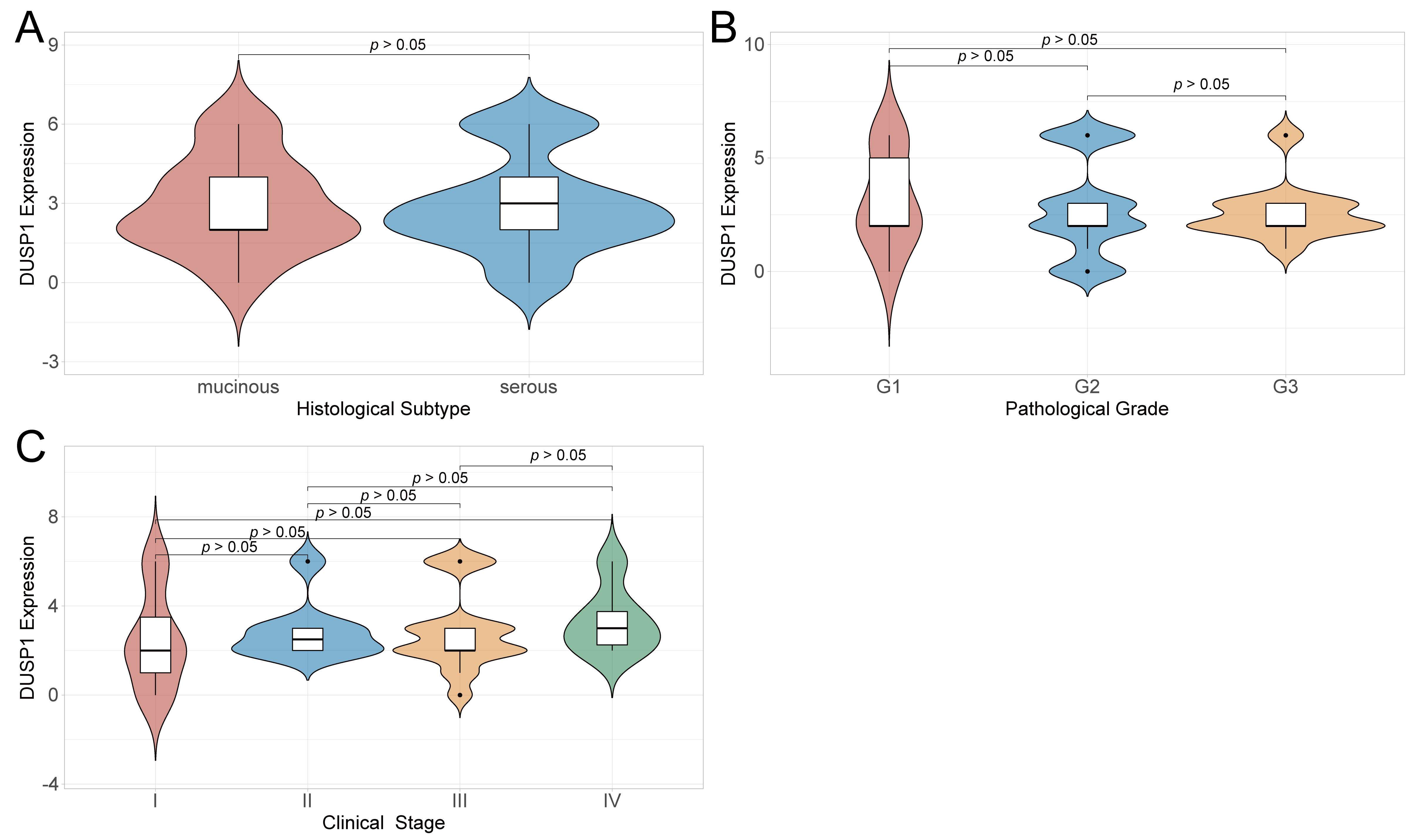

Supplement: Supplementary file 4 [file Image4.PNG]

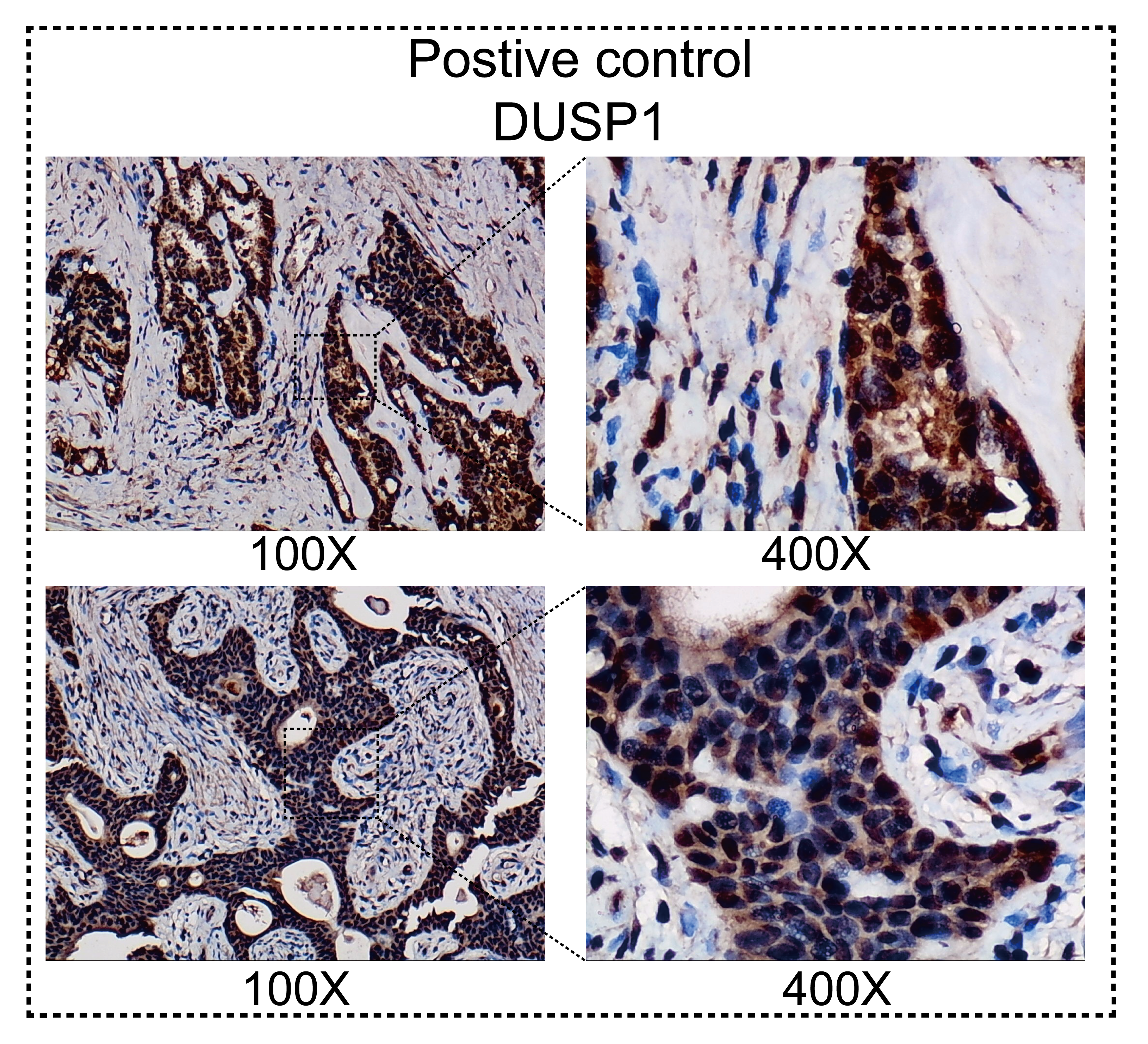

Supplement: Supplementary file 5 [file Image2.PNG]

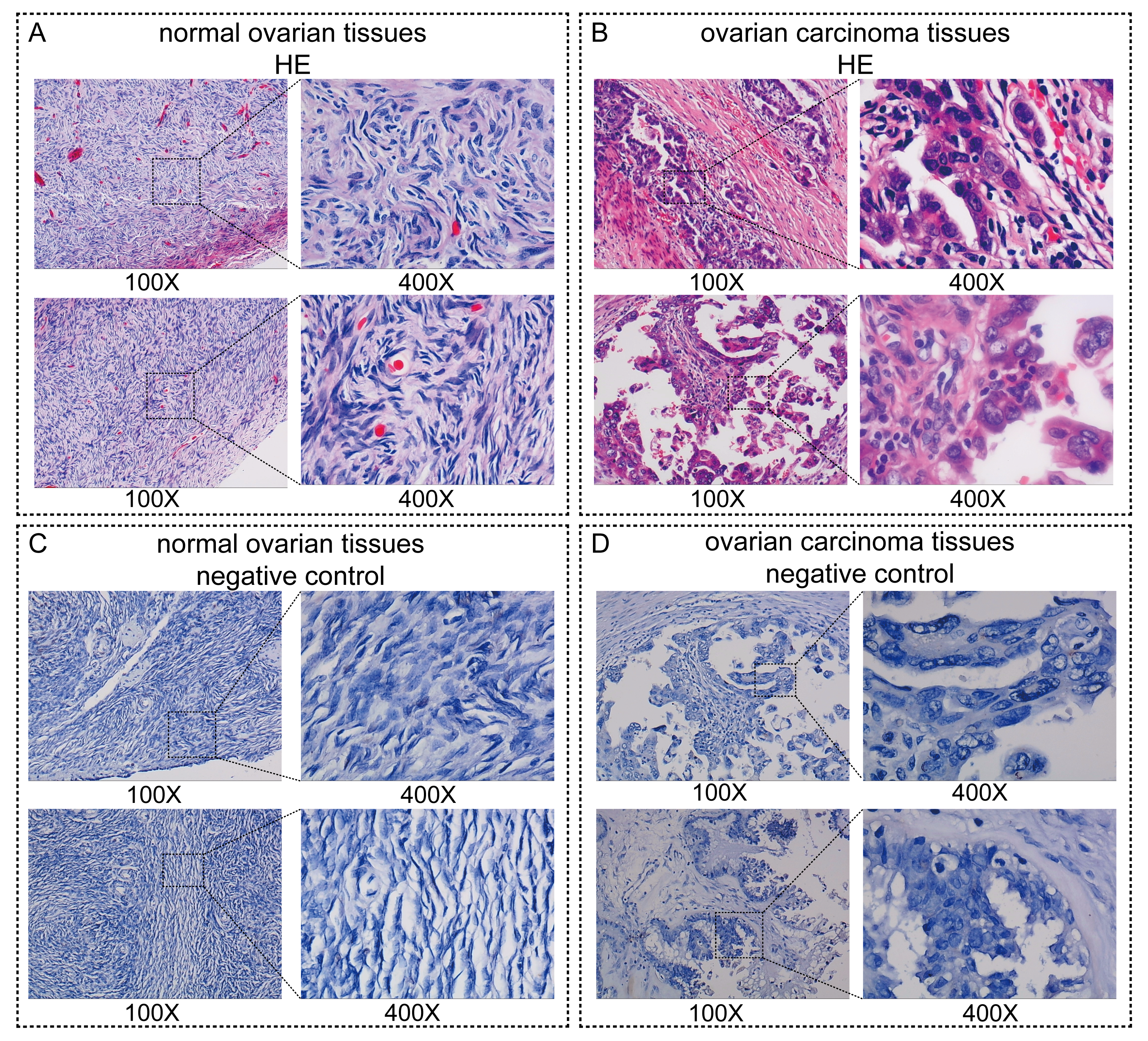

Supplement: Supplementary file 6 [file Image1.PNG]
